# Rationally designing antisense therapy to keep up with evolving bacterial resistance

**DOI:** 10.1371/journal.pone.0209894

**Published:** 2019-01-15

**Authors:** Seyfullah Kotil, Eric Jakobsson

**Affiliations:** 1 Program in Biophysics and Computational Biology, University of Illinois at Urbana-Champaign, Urbana, Illinois, United States of America; 2 Beckman Institute for Advanced Science and Technology, University of Illinois at Urbana-Champaign, Urbana, Illinois, United States of America; 3 Department of Molecular and Integrative Physiology, University of Illlinois at Urbana-Champaign, Urbana, Illinois, United States of America; Universidad de Santiago de Compostela, SPAIN

## Abstract

Antisense molecules used as antibiotics offer the potential to keep up with acquired resistance, by redesigning the sequence of an antisense. Once bacteria acquire resistance by mutating the targeted sequence, new antisense can readily be designed by using sequence information of a target gene. However, antisense molecules require additional delivery vehicles to get into bacteria and be protected from degradation. Based on progress in the last few years it appears that, while redesigning or finding new delivery vehicle will be more difficult than redesigning the antisense cargo, it will perhaps be less difficult than finding new conventional small molecule antibiotics. In this study we propose a protocol that maximizes the combined advantages of engineered delivery vehicle and antisense cargo by decreasing the immediate growth advantage to the pathogen of mutating the entry mechanisms and increasing the advantage to the pathogen of antisense target mutations. Using this protocol, we show by computer simulation an appropriately designed antisense therapy can potentially be effective many times longer than conventional antibiotics before succumbing to resistance. While the simulations describe an in-vitro situation, based on comparison with other in-vitro studies on acquired resistance we believe the advantages of the combination antisense strategy have the potential to provide much more sustainability in vivo than conventional antibiotic therapy.

## Introduction

In considering the possibility Antibiotic resistance is a major and growing health problem in the United States and around the world, due significantly to overuse of antibiotics in both humans and agricultural animals.[1] Because of the persistence of antibiotic concentrations in human and agricultural waste, and ultimate transition of those antibiotics into the environment, wild populations of bacteria are]] exposed to selection pressure for antibiotic resistance. [2] It has been estimated that the annual cost of antibiotic resistance to the US economy is between 20 billion and 35 billion dollars.[3] Although novel antibiotics are being discovered, the rate of discovery is not sufficient to overcome the rate of resistance development. [4] It is projected that a fundamental shift in antibiotic therapy will be occurring from broad spectrum natural products to narrow spectrum products, which might be synthetic or engineered as well as natural. [5] New antibiotics must be designed to overcome the fundamental mechanisms by which bacteria mutate to resist antibiotics. [6] Discovery of new antibiotics involves screening of naturally present biological products, which requires a lot of time and money. [7] Thus potential ways of rationally and rapidly designing antibiotic agents are important.

A prospective candidate for rapidly designable antibiotics is antisense molecules, which are nucleic acid analogues that base pair with its targeted mRNA (sense strand) to impede translation.[8] Design of antisense molecules is relatively easy, and earlier works have investigated this problem. Knowledge of the target gene’s sequence reliably predicts the binding efficiency between antisense molecules and its target. [8] [9] [10] In-vitro, several antisense-based drugs are already in development as antibiotics. Phosphorodiamidate morpholino oligomers (also known as morpholinos or PMOs), Peptide-conjugated phosphorodiamidate morpholino oligomers (PPMOs), [11] [12] [13] [14] [15] [16] and peptide nucleic acids (PNA). PNAs are shown to decrease the resistance of MRSA by hitting the MecA gene [17] [18]. In another approach, RNA-guided nucleases (phagemids) are shown to be selective at killing virulent bacteria and improving survival of infected animals [19] [20].

On balance based on progress in the last few years it appears that, while redesigning or finding new delivery vehicles will be more difficult than redesigning the antisense cargo, it will perhaps be less difficult than finding new conventional small molecule antibiotics. [21] [22]

**Fig 1:**
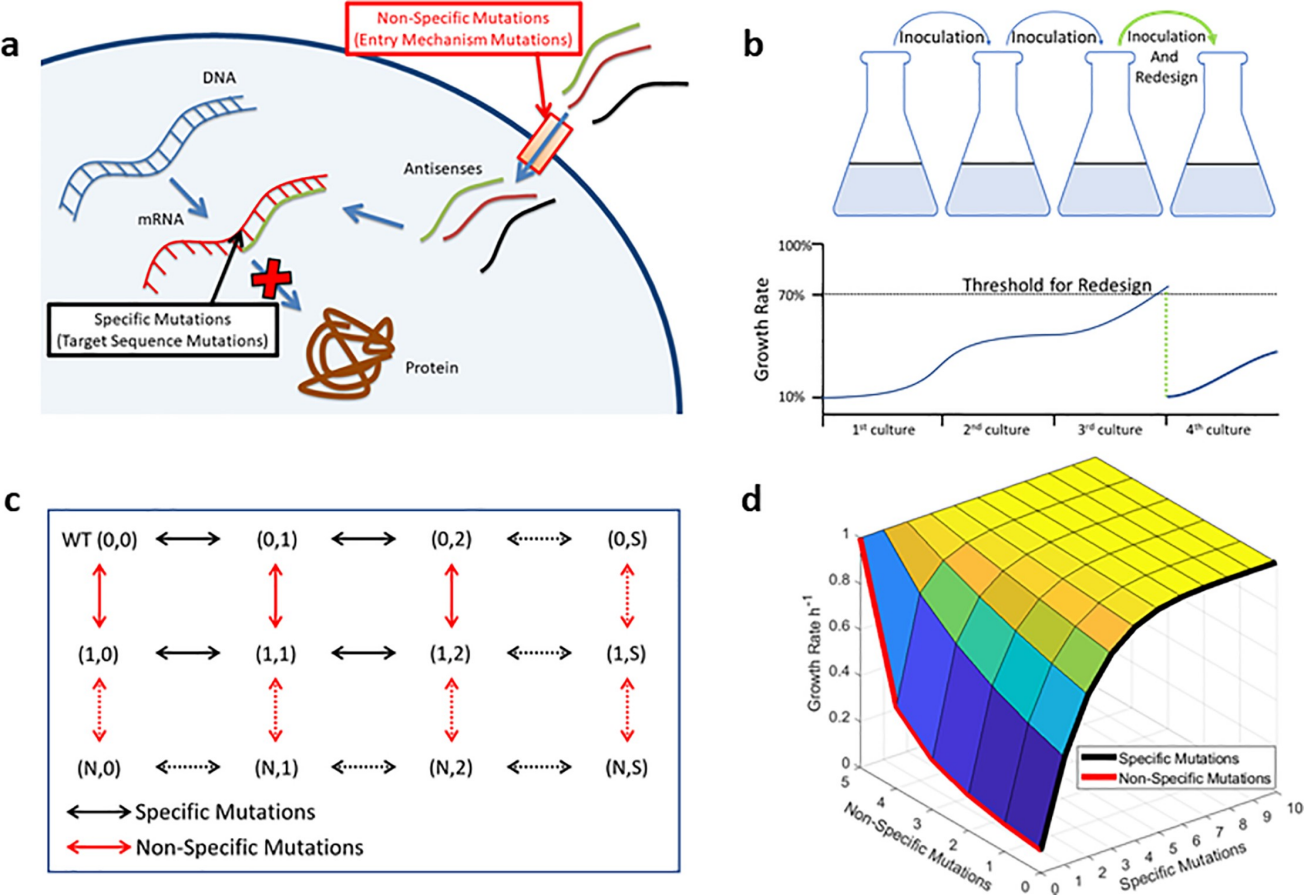
**Kinetic Diagram of Subspecies mutations**: **a**. Mechanism of action for antisense molecule. To alleviate its effects, there are two kinds of mutations: 1) specific mutations, nucleotide change in the target sequence, these mutations would only affect a “specific” antisense sequence. 2) nonspecific mutation, disruption in the entry mechanisms of antisense molecules, these mutations would nullify any antisense in “nonspecific” manner. **b**. Simulated system is bacteria grown in liquid culture. Computational experiments started with inoculated bacteria to a fresh medium containing antisense molecules. Growth continued until early stationary phase. Grown culture is used to transition to another medium. Serially passaged bacteria ensure continued evolution. After sufficient time, bacteria gain resistance to the therapy (growth rate surpassed a set threshold), at that time antisense therapy is redesigned. This action may or may not restore therapy effectiveness. If not, then therapy finalized. **c**. The kinetic diagram represents the mutational pathways. First, mutations in the targeted sequence (specific mutations, horizontal direction). Second, mutations that prevent entry of antisense into the bacterial cell (nonspecific mutations vertical direction). The direction of horizontal change is from left to right for acquiring specific resistance mutations. The direction of vertical change is from top to bottom for loss of delivery mechanisms. **d**. shows an example of a constructed fitness landscape. The height of the surface represents the growth rates associated with a kinetic diagram. The black edge represents the growth rates for specific mutations with all entry mechanisms kept intact. The red edge represents the growth rates associated with failing of entry mechanisms with 0 specific mutants. These two edges are used to derive all other growth rates, see methods for details.

Although principles of antisense therapy are studied, its applicative potential is vast and in need of further study. In this paper, we investigate the possibility of using antisense therapy to keep up with evolving resistance. We note that bacterial mutations in the target gene (which we call “specific mutations”, since the resistance will be specific to an antisense) can be countered relatively readily by redesigning the antisense sequence, but mutations that frustrate the entry mechanism (which we call “nonspecific mutations”, since the acquired resistance blocks all possible antisense) are not practically rescuable, Fig 1A. Unfortunately, experiments for induced resistance against PPMOs and PNAs shows mutations on inner membrane transporter, sbmA, which transports PPMOs and PNAs [23] [24].

On the face of it, the bacteria will preferentially evolve resistance to the entry mechanism—at least for therapy consisting of a PPMO and PNAs. In this paper, we suggest a therapeutic protocol in which bacteria will be induced into evolving resistance preferentially by specific mutations (readily recoverable by rapid redesign) versus non-specific mutations (not readily recoverable). Since the outcome of evolution depends on differential fitness between the mutational options, specific and non-specific mutations, then increasing the relative advantage of specific mutation versus non-specific mutation should prolong therapy effectiveness. We investigate this differential fitness with the formalism of constructing a ‘fitness landscape’. Previous studies have widely used fitness landscapes to predict and explain evolutionary trajectories [25] [26] [27] [28] [29]. Our goal is to manipulate the fitness landscape to alter the evolution to a less sustainable (from the perspective of the pathogen) course. Fitness landscapes used in present study minimally considers clonal interference. For each mutant, only permitted competition is among single specific and single nonspecific mutations. In the present paper, in which we will describe simulations of an in vitro experimental protocol with intense sustained antibiotic selection pressure, the effects of a multifactorial fitness landscape are minimized. In vivo, multifactorial fitness factors would be more relevant.

We evaluate the therapy in a simulated *in vitro* environment similar (but not identical) to a “morbidostat” [30]. In such an environment resistance is acquired much more rapidly than in the “hit-or-miss” transmission of resistant bacteria between hosts in the outside world. For example, in the morbidostat bacterial populations acquire essentially complete resistance to trimethoprim in a few weeks [31]. By contrast this antibiotic, introduced clinically in 1962, is still widely used and broadly effective, although some wild bacterial pathogens have acquired varying degrees of resistance to it [32] [33] [34], It is, of course, this dramatic acceleration of resistance acquisition in a population that may be totally characterized that makes environments like the morbidostat so valuable in studying resistance. [35]

## Model overview

The computational modeled system is equivalent to serial passaging of bacteria through a liquid medium containing antisense agents, Fig 1B. The computational experiment starts with inoculation of bacteria into fresh medium. In the medium, bacteria grow until the late exponential phase. Subsequently, the grown culture is used to inoculate a new medium. During serial passaging, the growth rate (maximum growth rate at exponential phase) of bacteria is monitored, in real-time. At the start of the experiment, bacterial growth rate was 10% of maximum growth rate (1 h^-1^, 41.5 min doubling time). As bacteria gains resistance mutations, the growth rate recovers to its maximum. The serial passaging is repeated until the growth rate of the bacteria stays below a threshold. We set the threshold at the 70% recovered of maximum growth rate. Once growth rate surpassed the threshold, then the sequence of the antisense-therapy is redesigned. Redesigning is aimed at decreasing the growth rate of the bacteria, opposing antisense resistance. New (redesigned) antisense can only counter the effects of specific mutations.

Serial transitioning, and redesign continues until redesigned sequences cannot rescue the therapy. That event is the therapy failure time. The goal of simulations is to compute therapy failure time for various therapeutic designs.

All simulations started with WT (most susceptible to therapy). WT can obtain two separate mutations: specific mutation (mutations on the targeted sequence) and nonspecificmutation (mutations to impede an entry mechanism for antisense). Likewise, each subtype can obtain the same kind of mutations. Mutational pathways of subtypes are summarized in the kinetic diagram, Fig 1C.

One simplifying assumption should be noted: there is only one first specific mutant, second specific mutant, etc., and there is one mutation that disturbs an entry mechanism. In fact, there can be multiple first specific mutants and multiple first nonspecific mutants and so on. A more completely detailed model would consider a web of mutations rather than a linear sequence of mutations. However, we believe the simplified model captures the essence of the competition of evolving pathogen versus evolving therapy. The choice of single mutation can be conceived to represent the worst possible (for the therapy) mutation among all possibilities. As a result, we assume that effects due to clonal interference will be minimal. Indeed, in-vitro experiments with high antibiotic selection pressure and well-mixing show minimal clonal interference [31].

Collection of all growth rates, associated with each subtype, determines the fitness landscape. A fitness landscape can be viewed as a surface: its heights are the growth rate, and its base is the kinetic diagram., Fig 1D. To determine the growth rates associated with the whole fitness landscape, we only needed to define the effect of specific mutations on growth rates. We gauged all other growth rates with respect to their ratio of functional entry mechanism to total entry mechanisms. Namely, we treated the effects of nonspecific mutations (entry-mechanism failures) as factors decreasing the intracellular antisense concentration, e.g. for setting with a maximum of two entry mechanisms, a mutant with one nonspecific mutation has half of WT’s intracellular antisense concentration.

As we will describe, we used two methods for searching the space of free variables of the system—possible growth rate values for specific mutations. First, we use randomly generated growth rates for specific mutations. Second, we use a parameterization for simplicity and ability to gauge specific mutation advantage (to bacteria), for details see Methods.

After defining the fitness landscape (all growth rates associated with mutants) we simulated trajectories of evolution. The dynamics of the simulations are carried out by a hybrid (deterministic and stochastic) model: growth is treated deterministically (by Runge-Kutta methods) whereas mutations are treated stochastically (by tau-leaping methods). Mutations are treated stochastically due to their rarity, for details see Methods.

## Results

Simulations are aimed to explore the space of possible specific mutation profiles versus a different number of entry mechanisms—one to five. We used two methods for searching the possible profiles of specific mutants.

### Randomly exploring the space of growth rates of specific mutations

**Fig 2:**
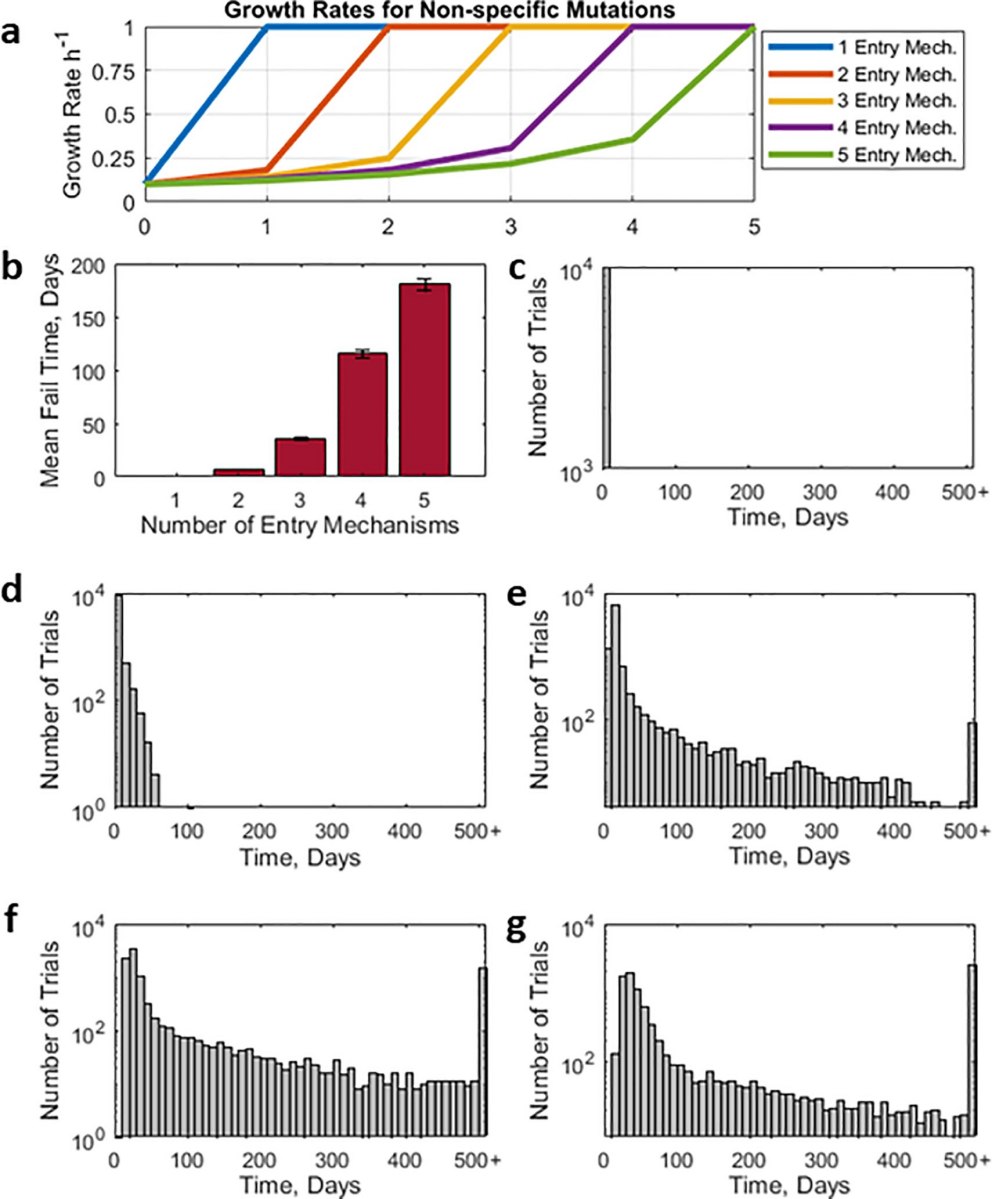
**Therapy Failure Time for Randomly Constructed Specific Mutants Caption: a.** Functional dependence of growth rate of pathogen vs number of mutations that inactivate entry mechanisms. Curves represent settings with maximum of 1,2,3,4, or 5 delivery mechanisms. **b.** shows mean-time of therapy failure for maximum of one to five entry mechanisms. Error bars are $\pm \frac{1\;standard\;deviation}{\sqrt{number\;of\;trials}}$ **c.** Histograms are number of computer experiments that resulted in indicated time to failure. **d**. shows the results for one delivery vehicles. **e.** for two. **f.** for three. **g.** for four. **e.** for five. Variables for effects of mutations on bacterial viability (as measured by growth rates) were chosen randomly, (by Method 1 as described in Methods Section) to span a wide range of possibilities. Bacterial generation time was 20 minutes. Simulated system was a monoculture with carrying capacity of 8x10^11^ bacteria. Total of 50000 computer experiments were done for 500 days, or time to failure, whichever came first.

First, we used random numbers for effect of specific mutations: the most general way of guessing the free variables. Details are described in methods as ‘randomly exploring free variables, method 1’. We sampled a total of thousand specific mutation profiles (a set of 10 growth rates). For same randomly selected growth profiles, we performed simulations with a different number of entry-mechanism, one to five. In other words, this method explores the generality of the effect of decreasing nonspecific mutation advantage by increasing number of different entry mechanisms. For example, if a therapy had only a single entry mechanism, and one nonspecific mutation, the intracellular antisense concentration will be zero and the therapy will fail. On the other hand, if a therapy had two entry mechanisms, and as before, had a one nonspecific mutation, the intracellular concentration will still be half of the intracellular concentration associated with WT and the therapy will continue. The greater the number of different entry mechanisms the less advantage to the bacteria for any single nonspecific mutation, as illustrated in Fig 2A.

For each fitness landscapes, we computed therapy failure time. Results are plotted in Fig 2B–2F. Mean therapy failure time increases with decreased nonspecific mutation advantage, Fig 2B. Associated distributions are given in Fig 2C–2F. For single and double independent entry mechanisms, the time to failure is comparable and low; with three independent mechanisms the time to failure is significantly extended. 5 independent mechanisms gave the best result. More independent mechanisms would undoubtedly have been better.

### Accounting for dependence of resistance on single or multiple mutations

It has long been known that a single mutation may render a bacterium totally, or almost totally, resistant to an antibiotic.[36] In a more recent experimental study that resembles our simulations, [37]acquired resistance to antibiotics was associated with multiple mutations. While it was not possible to infer how much each mutation contributed to the total level of resistance achieved, it could be inferred that each individual contribution was variable depending on which mutation it was and which antibiotic was used as the driver of the evolutionary change. In order to represent the effects of this complexity in the simplest possible way, we introduced the variable *ω* into our formulation. This variable represents the degree to which each single mutation will confer resistance. If *ω* is positive and large, only one or a small number of mutations is sufficient to enable the bacteria to escape the treatment. If ω is negative and large, then a greater number of mutations is necessary to enable escape from the treatment. Characterization of specific mutation advantage by a single parameter (ω) boils down the behaviour of simulations into two factors—the second factor is the advantage of nonspecific mutation (gauged by a number of entry mechanisms).

This formulation is also motivated by three additional factors: First, the function is fixed at 0.1 for 0 mutations and 1 for 10 mutations (boundary conditions). The maximum number of mutations were selected to be sufficiently high so that boundary effects would be minimal. Second, the function is symmetric around the linear line between the boundaries. Third and most importantly, the function is an exponentially saturating function which reflects diminishing returns. [38] [39] [40] Details of the explicit form of the function, (Eqs 4 and 5), is exhibited in methods as ‘exploring free variables by parameterization, method 2’.

**Fig 3:**
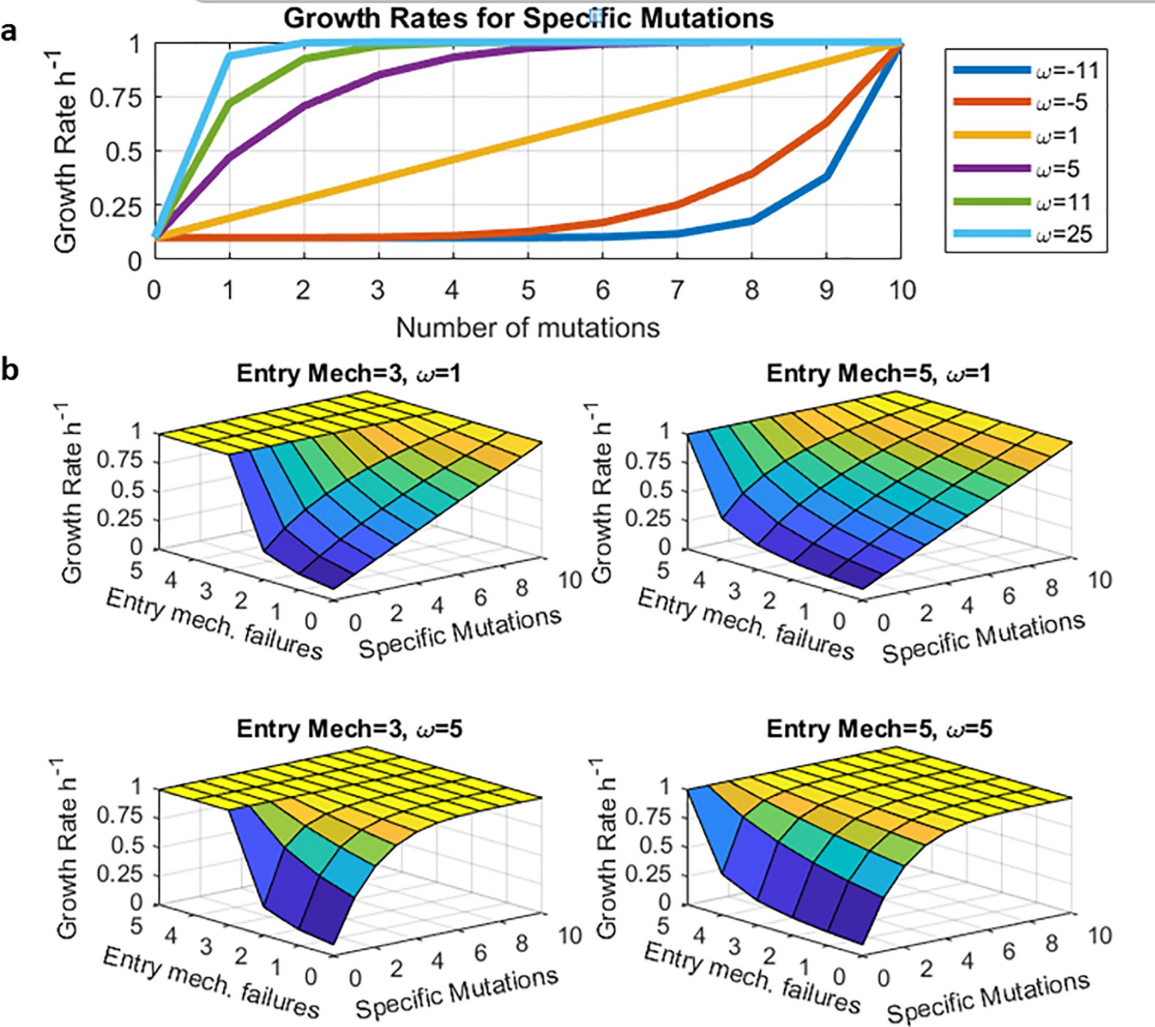
**Illustration of fitness landscapes for Different** ω: **A**. Functional dependence growth rate vs number of specific mutations as a function of the parameter ω in (Eqs 4 and 5). The higher the ω the more advantage the specific mutations provide. **B**. Sample fitness landscapes are made by combining curves from A. and Fig 2A. by (Eq 3). These surfaces define possible mutational pathways of evolutionary escape of the pathogens from the antisense therapy.

To show how fitness landscapes depend on *ω*, we have plotted growth rate profiles for specific mutants (with intact entry mechanisms) for *ω* equaling to -11,-5,1, 5, 11, 25, in Fig 3A. Example of fitness landscapes are given in, Fig 3B.

**Fig 4:**
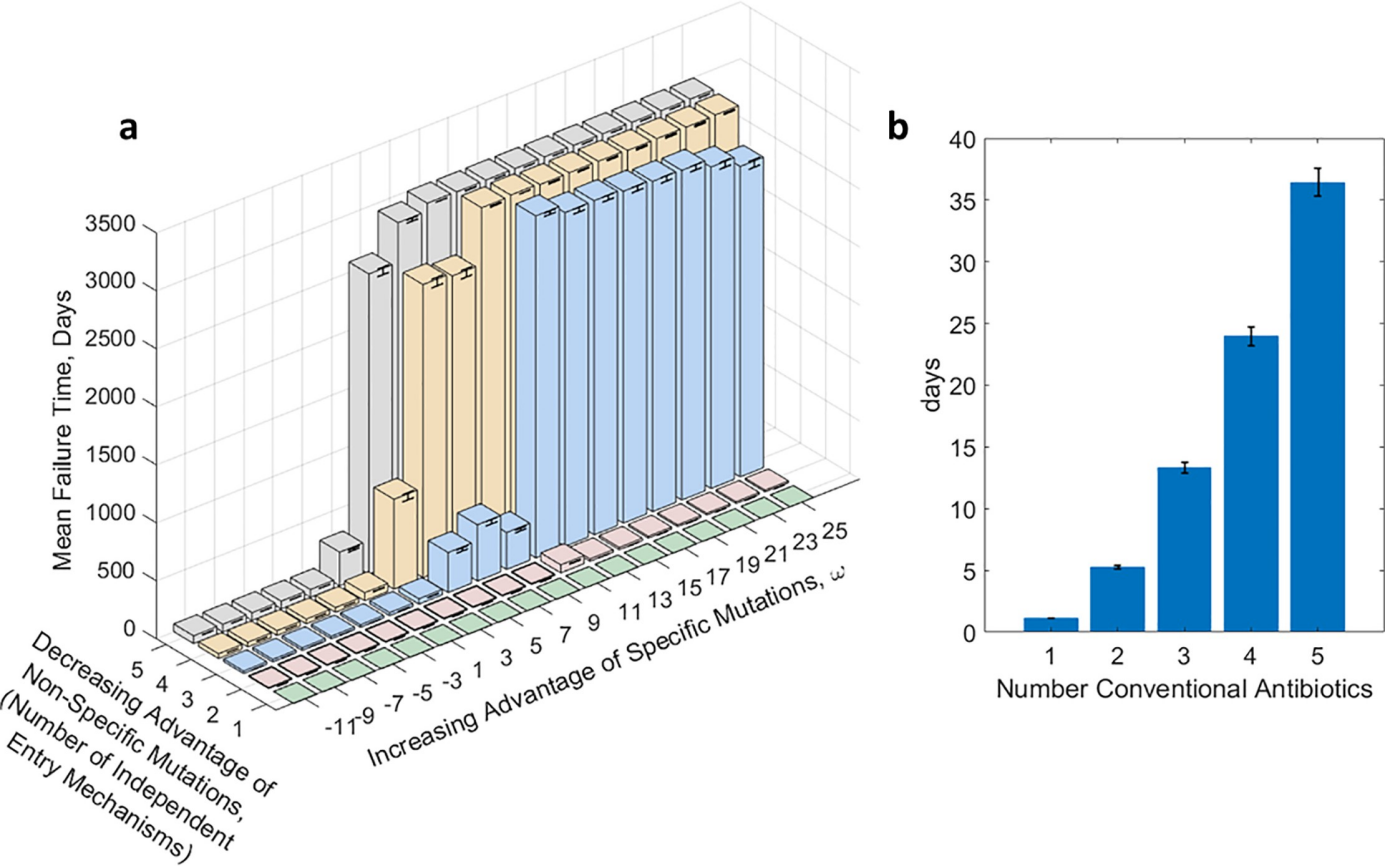
**Mean Therapy Failure Time for Simulations Made in 100 ml Rich Medium Caption: A.** shows summary of 1000 trajectories at each of the 90 combinations of possible independent-delivery-vehicle (5) and different values of ω (18), (or 12500 total) of which Figs, D, E, and F in S1 File show individual examples. It is seen that mean time to failure of the therapy is lengthened at higher ω and larger number of independent delivery mechanisms. In other words, making nonspecific mutations less advantageous and making specific mutations more advantageous elongates therapy efficacy. The simulations were performed as if the experiment were conducted in 100 ml rich medium **B.** shows mean therapy failure times for up to five-fold combination therapy of conventional antibiotics. Each data point is the mean of 1000 individual simulations. Under therapy the WT is assumed to have 10 percent of maximum growth rate and single mutation renders effectiveness of an antibiotic nil. Mathematically the simulation parameters are equivalent to our earlier simulations except in this case without any specific mutations. Error bars are $\frac{1\;standard\;deviation}{\sqrt{number\;of\;trials}}$.

After establishing a way for parameterizing the advantage of specific mutations, we compared the pair-wise effect of the specific mutation advantage and the nonspecific mutation advantage. We varied ω from -11 to 25 by increments of 2 and varied the number of entry mechanisms from 1 to 5. Results of mean time to failure are illustrated in Fig 4A. For any ω, mean time to therapy failure increases with increasing independent entry-mechanisms. For single and double entry mechanisms the mean time to failure is very low. The biggest jump happens from 2 independent entry mechanism to 3. After 3 independent mechanism, the trend is consistent and the mean failure time increases. This suggest that, an effective therapy should have at least 3 independent mechanisms and the specific target should be chosen such that ω is high. How to make such choices is described in the Ph.D. thesis of one of us (Kotil) and will be the subject of another paper. Examples of trajectories that went into the Fig 4A are presented as Figs C. D, E, and F in S1 File.

## Comparison with conventional antibiotics

To compare the timescale of antisense resistance, we simulated a case of conventional (small molecule) antibiotics. We simulate therapies having one to five independently-acting antibiotics. We assume that a single mutation can render an antibiotic ineffective: just like our assumption for entry mechanisms for antisense therapy.

As seen in Fig 4B, simulated mean time to failure ranged from approximately one day for a single antibiotic to 38 days for five antibiotics. In these simulations we assumed a single mutation would suffice to induce resistance to each antibiotic. A mathematically equivalent simulation would have been to assume a single antibiotic but to vary how many mutations would be necessary to confer resistance to that single antibiotic. The core of the simulation was—how long would it take for n resistance-inducing mutations to accumulate, where n varied from 1 to 5? Relevant experiments in which resistance to several antibiotics, one at a time, is induced in a bacterial population show time to failure of the antibiotic of six to ten days. [31] Even more rapid resistance is reported by Zhang et al.[41] Based on our simulation results, and assuming that our assumptions for mutation rates and generation times are similar to those pertaining for the experiments reported in Toprak et al,[31] that would suggest accumulation of 2 to 3 independent mutation events necessary for the antibiotic to fail.[42] We note that even in cases where additional mutations are needed the first mutations confer a significant resistance.

It is vital to consider the discrepancy between in-vitro and in real-life therapy efficacy. For example, in-vitro, bacteria gain resistance to an antibiotic in a matter of hours to weeks. However, in real-life, the same antibiotic stays efficient for decades. The selection pressure can be made much stronger, and therefore the acquisition of resistance much more rapid, in the laboratory than in an uncontrolled environment.

### Analytically solvable “toy model” illustrating central point of paper and relaxing assumptions

We constructed a simplified analytical model to capture the essence of above simulations. Our goal is to show the generality of our simulations and relax some assumptions, i.e. we relax redesign efficiency which we define as ability to spot the need to redesign and its execution. In our earlier simulations redesign efficiency was effectively infinite.

**Fig 5:**
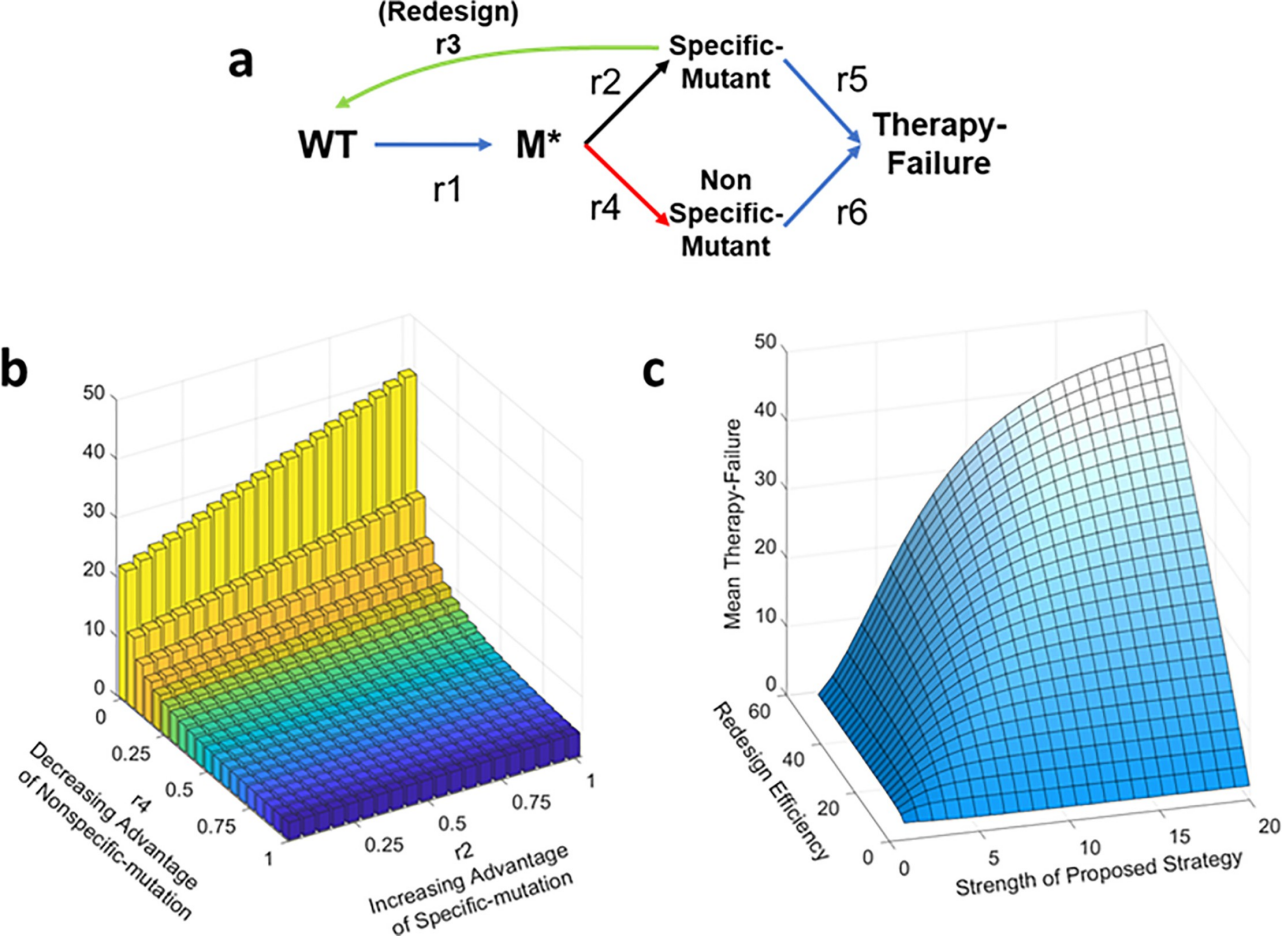
Simple analytical model: This figure shows a simple continuous-time markov model that illustrates our concept. A. Five states are considered, vulnerable (originally wild type), Mixed state of WT majority and its mutants (M*), resistant to antisense (specific mutant) resistant to delivery vehicle (nonspecific mutant), resistant to both antisense and delivery vehicle (therapy-failure). Rate coefficients for transitions are as shown. B. Plot of mean therapy failure time for r2 and r4. The plotted surface is associated with equation B in S1 File). r1, r5, r6 are taken as 1, since they are proportional to mutation rate, and we scale the mutation rate to be 1. C. Plot of mean therapy failure time for strength of proposed strategy (r2 maximized over r4) and redesign efficiency. r2 was parameterized by PS (proposed strategy). r4 was parameterized by 1/PS. R3 was parameterized by RE (Redesign efficiency). The plotted surface is associated with equation C in S1 File.

The model is a probabilistic model; see S1 File for complete details. Here we postulate five distinct states: WT (Wild-type), M* (mixed state, the both mutants, specific and nonspecific mutants, are present but in low abundance as background of WT bacteria), specific mutant, nonspecific mutant, and therapy-failure. Transitions associated with those states are given in Fig 5A. This model is the simplest model that considers three key variables. First, increasing advantage of specific mutations: modeled by increase in r2. Second, decreasing advantage of nonspecific mutations: modeled by decrease in r4. Third, variability of redesign efficiency: modeled by r3. Our goal for this model is to analytically solve for the mean time to reach therapy failure, Equation A in S1 File.

We investigate two modalities. First, we consider a case equivalent to our earlier simulations. Specifically, we investigated the interplay of increasing advantage of the specific mutations and decreasing advantage of the nonspecific mutations at the limit of infinite redesign efficiency, namely we computed the mean time to arrive at therapy-failure for increasing r2 and decreasing r4 at the limit of r3 going to infinity. Analytically, we solve for the mean time to arrive at state of therapy-failure, and then take the limit of r3 going to zero. After simplifying assumptions, we arrive at equation B in S1 File; the function increases with r2 and 1/r4. Indeed, the therapy failure time increases with the both components, increasing advantage of specific mutations and decreasing advantage of nonspecific mutations, Fig 5B.

Second, we analytically solve for the therapy failure time for the regime of non-perfect redesign efficiency versus the strength of the proposed strategy (maximize r2/r4). For modelling the amplitude of the proposed strategy, we model the increasing advantage of specific mutations and the decreasing advantage of nonspecific mutations with one parameter, PS (proposed strategy). Specifically, we parameterize r2 as increasing function of PS(r2 = PS), and r4 as decreasing (r4 = 1/PS). On the other hand, the redesign efficiency is basically r3. Again, after the same assumptions, we arrive at the equation C in S1 File; the function is increasing in RE (redesign efficiency) and PS (proposed strategy). Moreover, strength of the proposed strategy is a saturating function; thus, the redesign efficiency is the bound of a very well-designed therapy. More importantly, except for very low redesign efficiency, for all other values the therapy effectiveness increases the therapy failure time.

Thus, analytical analysis shows two principles: therapy failure increases with the specific mutation advantage maximized over the nonspecific mutation advantage, as well as, our design principle is valid regardless of redesign efficiency.

## Discussion

We have shown that an antisense therapy holds the potential to provide a sustainable therapy against the changing bacterial population, provided it is combined with a carefully designed strategy involving multiple different delivery vehicles.

Sustainability is due to relative ease of redesigning a new, effectively binding antisense to counter mutation of the target sequence in bacteria. However, we show that this potential is at risk by mutations that impair the entry mechanism of the antisense, as for example previously cited where mutations in protein sbmA impair the entry of PNAs and PPMOs. The situation of failed entry mechanisms is not as readily rescuable as the failure of antisense hybridization. Therefore, for a sustainable therapy, the bacterial resistance must arise from mutations on the target sequences rather than the entry mechanisms. Bacteria will gain resistance to any therapy; a major point of this paper is to devise and simulate a strategy to direct the bacterial resistance to preferentially mutate against the antisense hybridization rather than against the delivery vehicle.

The core concept is that the evolution selects its direction among its options to maximize the immediate advantage for resistance. As in our model, if we assume that there are two components for the fitness landscape, then evolution can only be directed if the advantage of the two components are not balanced. Our simulations showed the same fact, namely the simulations with minimized advantage of the entry-mutations and maximized advantage of the sequence-mutations exhibited the longest effective therapy. Counter-intuitively, attempting to minimize the resistance emergence for both components is inferior to focusing on the one that is harder to redesign. The study suggests a strategy for winning the “evolutionary arms race” against bacterial resistance [27] [42].

Since we are simulating an in vitro experiment with sustained antibiotic pressure, the time scales of our results are much shorter than the time scales of development of resistance in the real world. A degree of antibiotic resistance that develops in decades in the real world is replicated in weeks in such an in-vitro environment [31]. If the “weeks-to-decades” scaling between in vitro to in vivo holds true, then the strategy we propose could be sustainable for a time on the order of centuries.

A variation on the strategy we simulate would be to introduce multiple antisense sequences simultaneously. This would have the effect of retarding the bacterial ability to evolve resistance by specific mutations, both absolutely and relative to nonspecific mutations. Based on our assumption of very efficient antisense redesign, and hitting just one target at a time, the strategy of introducing multiple antisense sequences simultaneously would not extend the time to failure of the therapy. However, if these assumption were substantially relaxed, as it could be in future work in antisense therapy design, the tradeoffs would become more complicated. The underlying principle would remain, namely that the therapy should steer the bacteria to acquiring resistance to that aspect of the therapy that can be most efficiently redesigned to keep up with bacterial evolution.

One good reason for using multiple difference antisenses would be to take account of the fact that pathways in living cells exhibit a high degree of degeneracy; that is, that there are multiple pathways that accomplish essentially the same function.[43] This appears to be a general feature derived from each organism’s history of evolution and adaptation and is reflected in the fact that when pathway redundancy has been searched for in microbes, it has repeatedly been found.[44] [45] [46][47] However a consideration of pathway redundancy is often not reflected in antibiotic therapy, which focuses on one drug vs. one target or a narrow range of targets.[48] This has been effective (although the effectiveness is now threatened by evolved resistance) but is based on a single dominant pathway being the key to the viability of the pathogen, which is more the exception than the rule for critically important pathways in the bacterium. With the reconstruction of bacterial metabolic networks, it is possible to readily identify redundant pathways to target.[49][50] By providing a ready path to hitting all redundant functionally equivalent pathways simultaneously, antisense therapy provides a plethora of new potential targets for antibiotic therapy; that is, those targets contained in redundant pathways.

While we believe that the relatively simple model presented in this paper captures many of the essential features of a proposed antisense therapy for bacterial pathogenesis, some of the simplifications are significant and the model needs to be elaborated in future work. Some of the most important simplifications are:

- Our model assumes that redesign of antisense is effectively immediate and redesign of delivery vehicles takes effectively infinite time. This is what leads to the proposed strategy of guiding the evolutionary path of the pathogens to preferentially acquire resistance to the antisense. In fact the redesign of the antisense will not be quite immediate as it will depend on sequencing the population of the pathogens, and the pathogens will not escape all along the same route; i.e., there is more than one mutation that will reduce the effectiveness of the antisense. Also, the pathogen may acquire resistance by developing a redundant pathway to the one hit by the antisense rather than by mutating the gene targeting the antisense. A discussion of other complexities of antisense design is given in Hoynes-O’Connor and Moon.[51] At the same time that antisense redesign may be more complex than we assume, it may also be that advances in entry vehicle design may make it more feasible than we postulate to redesign entry vehicles. The broad principle of directing the pathogen evolution in the direction to which we can respond most readily will always pertain, but the tradeoffs between antisense and entry vehicle will be more complex than we show in this paper.
- Our model takes very limited account of the heterogeneity of the pathogen genome. Different variants of the same pathogen will vary in significant ways. A more comprehensive and complex model would take this into account.
- Current antibiotic strategies generally target metabolic or signaling pathways that do not have redundancy. However we now realize that most pathways are redundant. Because of the relative ease of designing antisense as opposed to developing new antibiotics, with antisense it should be possible to simultaneously hit redundant pathways, opening up a wide range of targets.

For the above reasons, the complete computational model that would ultimately help to guide antisense antibiotic strategies would be significantly more complex than the model presented here. However we believe the model presented here can provide a useful framework on which to build the full complexity of the “arms race” between evolving antibacterial strategies and bacterial evolution itself.

Of course, the in vitro experiment we simulate is very simplified compared to the real world. To comparably simulate the real world the therapy strategy and the evolutionary properties of the pathogen would need to be embedded in an agent-based model of spread of an infectious agent in a population.[52] Agent-based population models of infection can be used to evaluate mitigation strategies.[53] Real-world implementation of the strategy suggested by our paper would imply continual testing and sequencing of samples of the evolving pathogen, together with redesign of antisense and redeployment of delivery vehicles to counteract developing resistance according to the steering strategy presented in this paper, with specifics guided by a frequently updated agent-based population model. Within a population model, clonal resistance might be expected to be more significant than in the in vitro case, since in the population the antibiotic therapy is but one of multiple selection pressures on the bacterial evolution.

In addition to combating infection by ingestion, antisense is potentially useful as an environmental disinfection tool.[54] It is also part of the toolkit for creation of a synthetic biology.[55]

In addition to infectious disease, antisense therapy is a promising approach to cancer treatment.[56] As do bacterial pathogens, so also cancer cells evolve as the disease progresses, [57] thereby acquiring drug resistance during treatment of an individual. Our mathematical analysis and simulation techniques should therefore also be relevant to designing antisense cancer therapy.

In a more complete analysis of our approach, the sequences selected for antisense would be chosen not only for effectiveness against the pathogen but also in order not to target important processes in commensal bacteria. It would be counterproductive to the health of the patient population to exert selection pressure on commensal bacteria with which we have a mutually beneficial relationship—a pressure which conventional antibiotics exert.

In addition to a mutation, the target pathogen could respond to the proposed therapy by conjugation; that is, by importing a replacement gene or even a complete operon with equivalent function from an organism of a different species in the host microbiome. Our proposed treatment, because of its specificity, would provide more selection pressure for such a bacterial response than a conventional antibiotic would. Although this could be combatted with continued sequencing and adjustment of the antisense, a particular advantage of the antisense would be compromised, since the redesigned antisense would now hit the commensal bacterium that donated the sequence to the pathogen, unless a totally different target in the pathogen were chosen. Considering this possibility points up the fact that, even with the best and most specifically targeted antibacterial tools, the war against infectious disease will involve a continual and perpetual arms race, rather than containing any prospect of final victory.

Our model is not sufficiently detailed as to consider either concentration of the antibiotic nor the efficiency of delivery. Rather both of these factors are rolled together into a single killing efficiency. A future model could include more detail.

As with all human interactions with biological systems, the system will respond in some other ways than only in the way intended by the human intervention. In particular, the approach outlined in this paper will drive the bacteria to different strategies from single mutations, which would be the easiest to redesign for. An alternative strategy for the pathogen would be to acquire equivalently functional genes or operons from commensal bacteria. Redesigned antisense could still be effective, but it would also be effective among some of the commensals. In general an effective strategy for pathogens would be to make themselves as similar as possible to commensals, or even to the host organism. This would require targeting strategies to be more specific, to zero in on the critical differences between the pathogens on the one hand, and the commensals and the host on the other hand. It also should be recognized that this approach, like all antibiotics, would modify microbial ecology. Like other microbes, pathogenic bacteria play a role (only incompletely understood) in microbial communities. Broad spectrum antibiotics modify microbial communities. The proposed specific approach would also modify those communities, albeit in a different way.

In the broad spectrum of biocides, the proposed approach would be notable for its specificity. Most biocides kill a broad spectrum of organisms. The proposed approach would aim to kill only one organism, and kill that highly effectively.

This work in principle would apply to any antibiotic with the properties of 1) specificity with respect to organism, 2) with effectiveness degraded by a single mutation or small number of mutations, and 3) rapidly redesignable to account for bacterial evolution in response to the drug. While antisense seems most obviously to fulfill these criteria, other approaches may emerge that do so as well.

## Methods

### Redesign

The redesign results in nullification of specific mutations: according to the Fig 1C, it is a move from (*i*,*j’s*) to (*i*,0). Qualitatively, each trajectory will start at the origin (0,0) and then become (0,1) then (0,2) and so on until bacteria has evolved passed the threshold for redesign. The redesign of the antisense sequences will make bacteria susceptible again, that is, (0,2) will become (0,0).

### Simulating growth and mutation dynamics

The rate equations that are linked to the kinetic diagram in Fig 1C, in the absence of any antibacterial intervention, are;

$$\frac{dB_{\left(i,j\right)}}{dt}=\frac{R}{K}∙B_{\left(i,j\right)}(t)∙\varphi (t)-{NMR}_{i,j}$$ (Eq 1)

Where the *B*_(*i*,*j*)_(*t*)′*s* are population size in dimensions of cell number, *R* is growth rate in dimensions of 1/hour, *K* is the carrying capacity in dimensions of cell number, *φ*(*t*) is the nutrient level in dimensions of cell number; nutrient amount is expressed in number of bacteria that could be made by that medium. *NMR*_*i*,*j*_ is the net mutation rate by which the strain (i,j) is altered to another strain.

In a closed system nutrient will be consumed as the bacteria grow, according to the relationship:

$$\varphi (t)=(K-\sum_{j=1}^{n}\sum_{i=1}^{n}B_{i,j})$$ (Eq 2)

Where *K* is the carrying capacity. The starting nutrient level is K minus the initial number of bacteria, which is very small; thus, in our system the starting nutrient level is *K* to a very close approximation.

One simplifying assumption should be noted: We consider the bacteria are only capable of taking a particular mutation against a particular antisense, e.g. there is only one first specific mutant, second specific mutant, etc., and there is one particular mutation that disturbs an entry mechanism. In fact, there can be different first specific mutants and different first non-specific mutants and so on. A more completely detailed model would consider a web of mutations rather than a linear sequence of mutations. However, we believe the simplified model we present captures the essence of the competition of evolving pathogen vs. evolving therapy. In the future, more advanced versions of the model to be used to guide therapy could take these differences into account. We also assume that effects due to clonal resistance will be minimal. This is indeed the case in in vitro experiments with sustained antibiotic selection pressure [33].

If we now introduce an antibiotic (antisense), the growth rate is modified according to the following relationship

$$R_{\left(i,j\right)}=\frac{r_{max}}{1+(\frac{r_{max}-R_{0,j}}{R_{0,j}}∙\left(\frac{N-i}{N}\right))}$$ (Eq 3)

*r*_*max*_ is the maximum growth rate of the WT, N is the number of different entry vehicles deployed at the beginning of the simulated experiment, and *i* is the number of entry mechanisms that have been lost to the bacterial ability to evolve resistance such mechanisms. If all of the entry mechanism have been neutralized then *N = i* and the total growth rate equals to maximum rate *r*_*max*_ h^-1^. The quantity *R*_0,*j*_ (Specific mutations) is computed by two methods as described below:

### Randomly exploring free variables, method 1

The degree of growth rate inhibition vs. specific mutation is computed with random numbers for every simulation run, as follows: For each run we select 10 random numbers in the range 0.1 to 1 (0.1 h^-1^ is the growth rate of WT under pressure from the therapy and 1 h^-1^ is the maximum growth rate attainable by bacteria), and order them from smallest to largest, *R*_*0*,*j’*_*s* (specific mutants). Upon the first mutation, the growth rate increases to the fraction represented by the first number. Each subsequent mutation moves the growth rate to the next number, up to ten mutations. This method provides us with un-biased, most general estimation of the *R*_*0*,*j’*_*s* (specific mutants).

### Exploring free variables by parameterization, method 2’

Constructing a mutation-sensitivity profile involves reducing the sensitivity profile to a single integer parameter *ω*. The relationship between bacterial growth rate and number of mutations is given by the equations

$$R_{0.j}=1-0.9\;{\left(\frac{\left(10-j\right)}{10}\right)}^{\omega },for\;j=0:10,\omega \geq 1$$ (Eq 4)

$$R_{0,j}=0.1+0.9\;{\left(\frac{j}{10}\right)}^{\left(-\omega \right)},for\;j=0:10,\omega <-1$$ (Eq 5)

#### Scale of the simulations and its hybrid (deterministic and stochastic) execution

In the simulated experiments, the starting point is a bacterial population of 1/20000 of the carrying capacity of the flask, which is taken to be 10 optical density (OD). The size of the vessel is 100 ml, giving 8x10^11^ bacteria as the carrying capacity. The initial population is thus 4x10^6^ cells. In order to do the entire simulation in the growth phase[30], there is re-dilution whenever a simulated flask reaches half of carrying capacity, or 4x10^11^ cells. This scale of the volume is selected to have a one-to-one comparison with in-vitro evolution experiments that are performed with conventional antibiotics[28].

The time scales for the two major processes of the simulation (growth and mutation) differ by a factor of approximately 10^10^; mutation is a much more rare event than faithful replication of a cell. Specifically, we assume a mutation rate of 5 * 10^−10^ per generation per nucleotide.[58] This difference in scales leads to different mathematical representation of the two processes. Growth is treated continuously and deterministically, while mutations are introduced stochastically (probabilistically). At each simulation step, first the growth portion of the system is simulated deterministically by a fourth order Runge-Kutta method, [59] then the cells that are divided are calculated and the mutants are computed analogous to tau leaping methods,[60] [61] by a Poisson random number with the rate of mutation. The time step is taken to be 60 minutes.

In addition to mutations, probability is also introduced when simulating dilution. In this phase of the simulated experiment, .9999 of the culture is discarded. Each subpopulation is represented by the appropriate fraction of the entire population superimposed on a Poisson distribution scaled according to the expected number of individual cells. For a dominant subpopulation the variance/mean ratio of the distribution is very small. However, for very small subpopulations, the corresponding ratio is quite large, so there is a chance of a significant fractional change in this population. Simulations were terminated after 3000 days (if not noted otherwise), if loss of efficacy of the therapy was not achieved earlier.

In our simulations, the concentration of antisense or antibiotics are always administered at a constant amount. This is made to escape from modelling concentration dependence of the agents, which would involve extra parameters.

## Supporting information

S1 File Detailed sample trajectories and mean hit-time calculations. (DOCX)

S2 File Data points for figures. This Excel file contains the data points for display items in the main body of the paper.
(XLSX)
