# Supplementary material for: Rationally designing antisense therapy to keep up with evolving bacterial resistance
Source: PLoS One. 2019 Jan 15;14(1):e0209894. doi: 10.1371/journal.pone.0209894 (PMC6333403; doi:10.1371/journal.pone.0209894)
Supplement: S1 File — (DOCX) [file pone.0209894.s001.docx]

Four trajectories from Fig 4 in the main paper are chosen to be presented in detail. Specifically, trajectories were selected from 3 entry mechanisms and $\omega$=1, 3 entry mechanism and $\omega$=5, 5 entry mechanism and $\omega$=1, 5 entry mechanism and $\omega$=5. The time course of these trajectories were represented with Muller Plots.

**Muller Plot**

The Muller plot is an effective way of visualizing evolutionary dynamics. [1]In Muller plots the y axis is the fractional population, the x axis is the time, and each mutant has its own color. At each time the fractional population of a mutant is represented by the fractional coverage of the graph by its color. For example suppose the total population is half WT and its mutant. Let’s assume WT is indicated with black and the mutant by white; then the half would be black and other half would be white. Another advantage is ease of illustrating the ancestor of a mutant. When a mutant arises, it originates from the color of its ancestor. An example trajectory is given in Fig A, for a very simple system. The population starts with wild type (WT)and there are linear mutations e.g. from WT to first, from first to second and so on. In our plots the minimum relative abundance to be represtend in the Muller plot is 0.01.


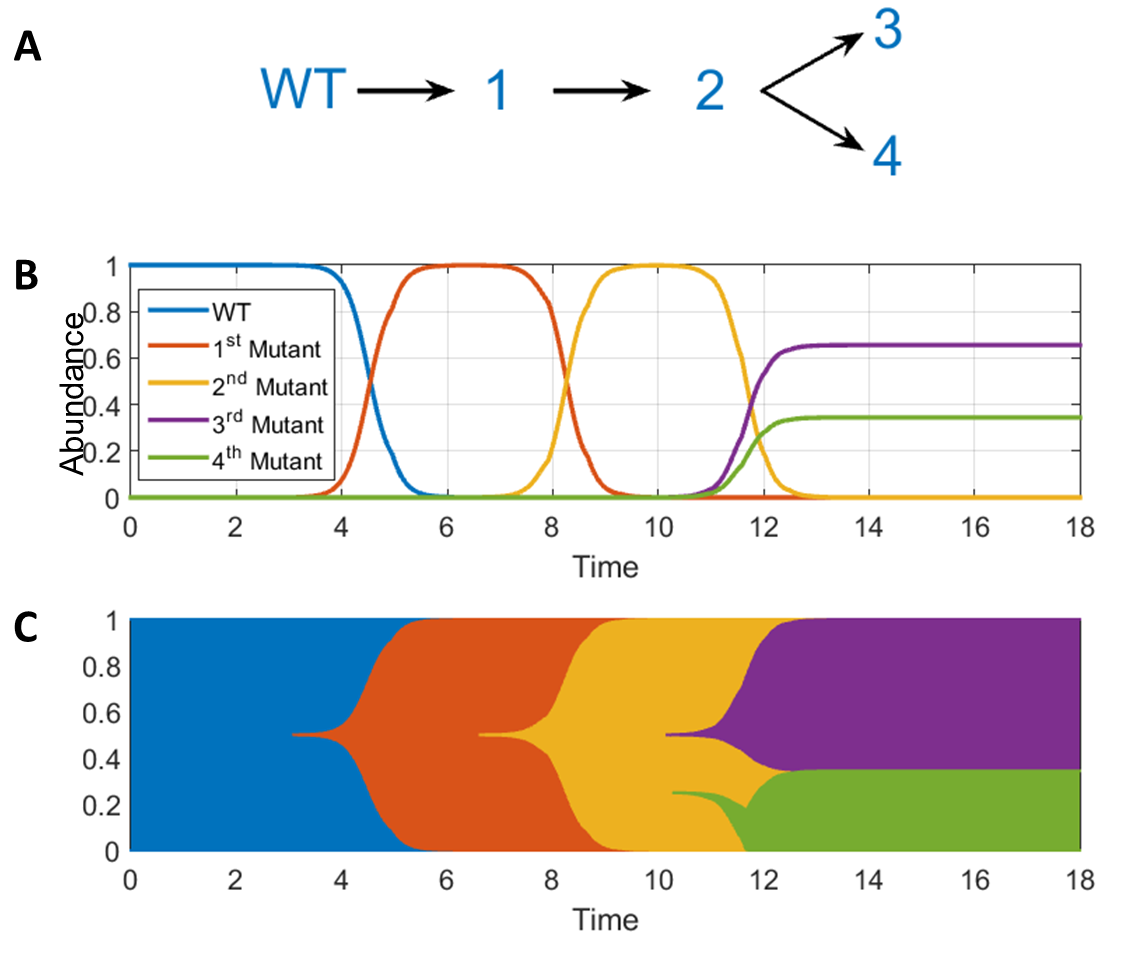


**Fig A: An Example of a Muller Plot*.*** Panel A. Simulated system as represented by the kinetic diagram; first mutant arises from WT, second mutant arises from first and third and fourth branches from second mutant Panel B. Line plot of population dynamics of mutants are presented. Mutations proceed in the order, 1^st^, then 2^nd^ , and then population shared by 3^rd^, 4^th^ . First mutant arises at 3 days, second mutant arises at 6.5 days, followed by third and fourth mutant around 10 days. The time units are days in this simulation. The same trajectory is also plotted as a Muller plot in Panel C. The blue indicates that entire population is WT at start of the trajectory. At 3 days first mutant arises from the WT and grows substantially until 6.5 days. At 6.5 days first mutant gives rise to second mutant and it dominates the population by 9 days, followed by third and fourth mutant arising around 10 days. The Muller plots’ advantage is ease of visualizing which mutant arises from which predecessors. However, it lacks the direct visualization of quantitative information that the line plot (B) provides. In this paper, we have used Muller plots for illustration where it is more important to emphasize the course of evolution and its origins. On other occasions, we plotted the line plot because it can be transformed to log scale, which enables the viewer to observe what happens in all scales.

**Color coding of Trajectories**

Fig B shows the color code to be used in the Muller plots in Figs C, D, E, and F. The wild type bacterium is represented by 0,0 in the upper left corner of the chart, while the lower right hand corner is the most resistant strain, having acquired 10 specific mutations against the antisense and non-specific resistance against all five available delivery vehicles. Non-specific mutations are given different colors and their specific mutants are made as their shades.

**
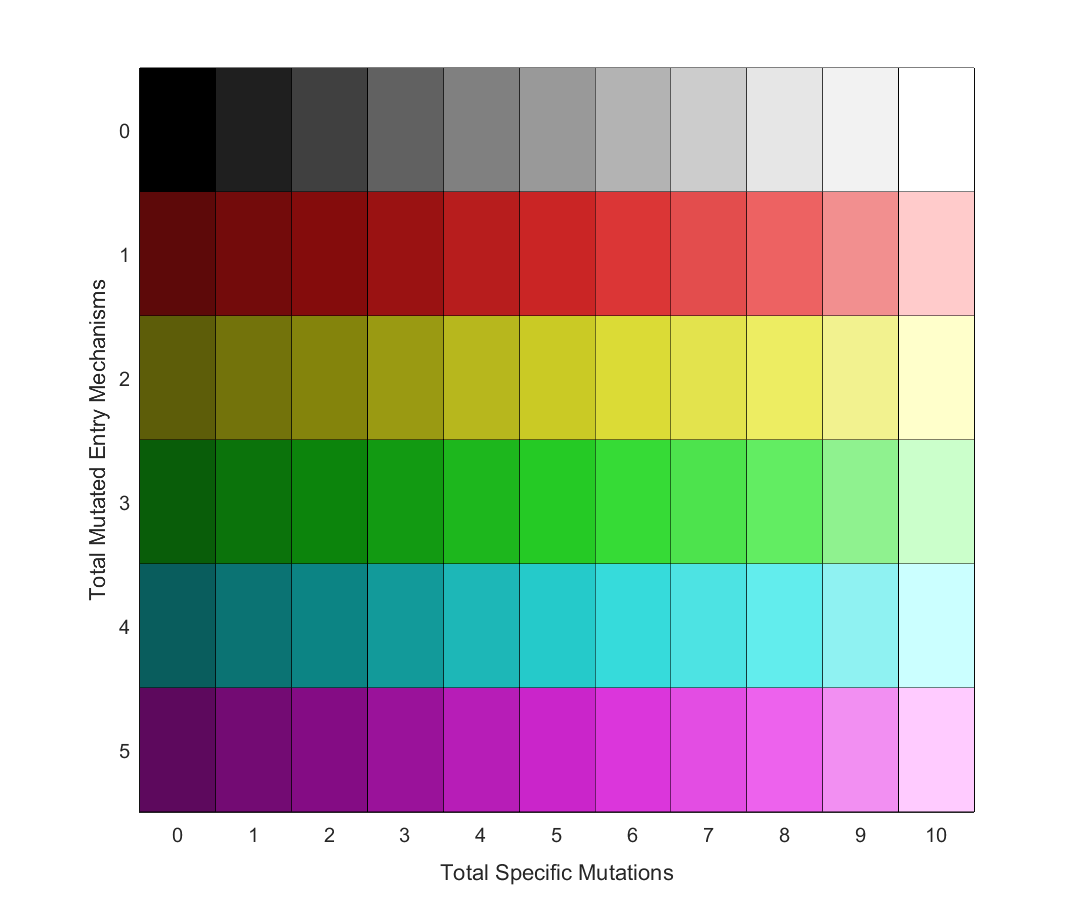
**

**Fig B.** **Color coding for the Muller plots.** Caption: We have chosen different color for number of mutated entry mechanisms and their shades for their specific mutants. Coordinates of a colored box is the corresponding mutant. For example the (5,10) is 5 entry mechanism failures with 10 specific mutatations, and its color is the brightest pink, lower right corner.

**Trajectories**

In Supplementary Fig C, the system is simulated with $\omega$=1 and with 3 independent entry mechanisms. The Muller plot is shown in Fig C, panel A. Through time WT is replaced by other mutants. When the population doubling time falls to a threshold of 56 minutes at 41.6 days, all of the specific-mutants (*B_i,j_* for all *j*  returns to *B_i,0_*) are nullified by redesigned antisense. Panel B shows the doubling time of bacteria. Panel C shows dynamics of bacterial populations with different numbers of entry mechanism failures. These trajectories are plotted in log 10 scale for y-axis. The log scale was chosen because this representation enables us to observe all of the scales at once, made necessary by the fact that the range of size of subpopulations varies by up to 10^13^. Mutants with one failed entry mechanism arise quickly but remain at a low level, until around 40 days when the second entry mechanism fails and takes over the population. Third entry mechanism failure follows quickly thereafter.


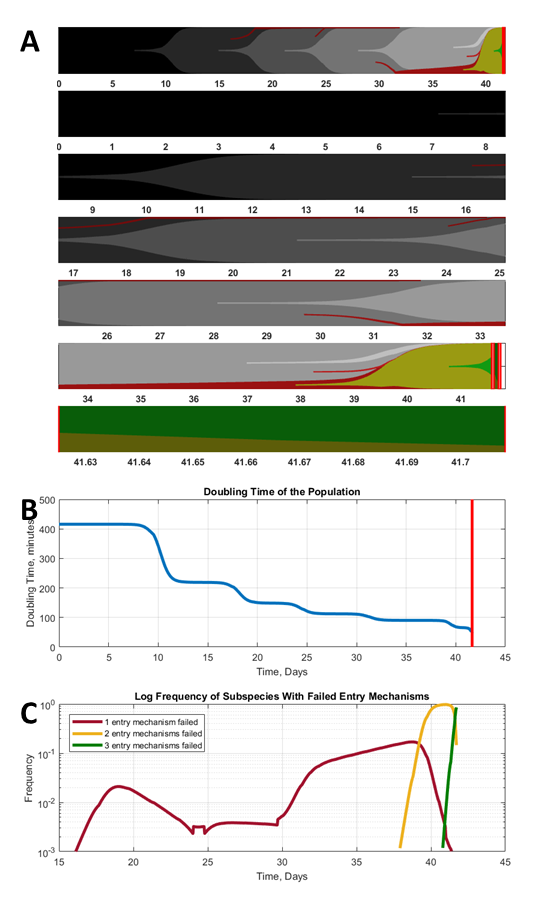


**Fig C:** **Sample Trajectory With Three Entry Mechanism and ω=1 Caption:** Panel A) The top panel shows the full trajectory. Last panel is the last redesign cycle (41.62 days to 41.8 days), and the panels in between are shorter parts of the full trajectory for more detailed inspection. Therapy lasts for 46 days. The vertical red lines mark the time of redesign. In this trajectory there is only one redesign at 41.62 days. Until failure we see that the first specific-mutant (0,1) takes over the population in 11 days. Then the second specific-mutant (0,2) takes over at 20 days. Then the third specific-mutant (0,3) takes over at 26 days. Before the third specific-mutant takes over, first entry mechanism failure is noticable on two occasions (mutants having relative abundance of 0.01 or more are represented) at 16th and 24^th^ days respectively. However the first two entry mechanism mutants die off by competetion. Right after third specific-mutant (0,3) dominates, the non-specific-mutant (1,3) arises from (0,3). In the meantime the fourth (0,4) specific-mutant also increases. At 33 days (1,3) and (0,4) comprise the most of the populations. The mutant (1,3) gives rise to second entry mechanism mutant (2,3) around 37 days. The (2,3) having very high growth rate takes over the entire population very rapidly and gives rise to (3,3). (3,3) increases slightly but the overall doubling rate exceeds the threshold for redesign. Redesign happens, there are two subpopulations at the start of the redesign (3,0) and (2,0), in very rapid time (3,0) increases for the full escape. The therapy can no longer be rescued by redesigning the cargo. All of the three entry mechanism have failed. Panel B) Doubling time versus time of simulation is plotted. The doubling time starts at 420 minutes per generation. The doubling time decreases step-wise (growth rate increases) as specific-mutants takeover. The threshold of 56 minutes is reached at 41.62 days. A cargo redesign is made at this time. Redesign is indicated with red vertical line. However the doubling rate does not recover since all off the entry mechanisms have failed and the therapy fails. C. Relative populations of entry mechanism mutants are plotted with log10 scale. The first entry mechanism mutant stays low but fluctuates. This mutant gives rise to second entry mechanism mutant which takes over at 40 days and paves the way for the final and third mutant. Third mutant arises at 41 days and dominates the population at 41.8 days. At this time the therapy halts.

In Fig D, trajectory of evolution is simulated with parameters $\omega$=1 and 5 independent entry mechanisms. Fig D panel A shows the Muller plot. It is seen that therapy lasts approximately 460 days. Total of seven redesigns are made. After 7^th^ antisense redesign two entry mechanisms fails, eventually third, fourth and fifth entry mechanism fail at 463.6 days. It is particularly interesting that the single entry mechanism mutant is not detectable before double entry mechanism mutant arises. This phenomenan of skipping an intermediate mutant has been called stochastic tunneling in the context of cancer genetics [2]. In our simulations, as in other systems, stochastic tunneling happens because the number of cells in the simulation is high compared to the reciprocal of the mutation rate. In Fig D, change of doubling time through course of the simulation is plotted. In Fig D, panel C, the entry mechanism mutants are plotted with log scale. This representation enables us to spot the stochastic tunneling that happened in this trajectory. The first entry mechanism mutant stays low but fluctuates. Many times the second entry mechanism mutates but each time dies out due to a small population extinction effect [3]. At 410 days the second entry mechanism mutant arises from the low single mutant and increases to take over the population. Eventually all of the entry mechanisms fail.


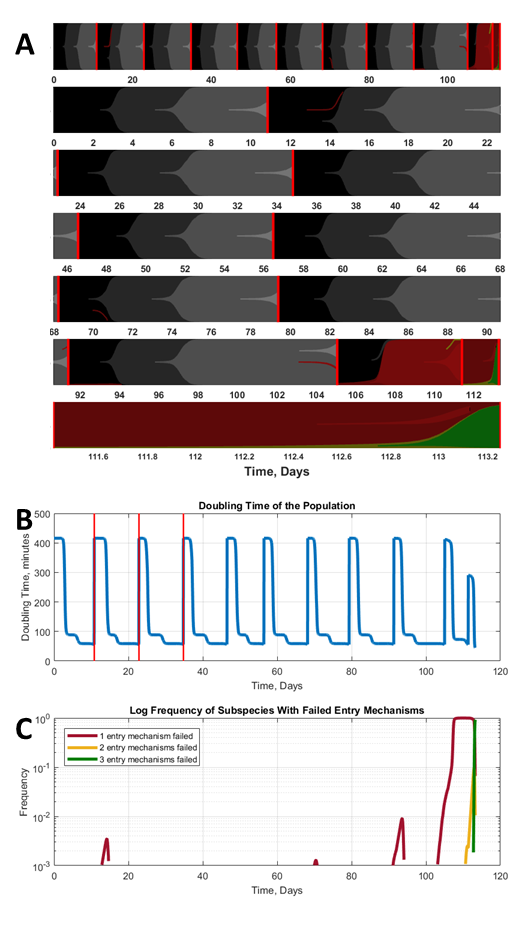


**Fig D*: Sample Trajectory with Five Entry Mechanism and ω=1***

A) The top panel shows the full trajectory. The last panel is the last redesign cycle (463.46 days to 463.66 days), and the panels in between are shorter parts of the full trajectory for more detailed inspection. The vertical red lines mark the time of redesign. Therapy lasts for 463 days. In this trajectory cargo is redesigned 8 times. Until first redesign we see (0,1), (0,2), (0,3), (0,4), (0,5), (0,6), (0,7) specific-mutants, dominating the populations at 11 days, 16 days, 26 days, 35 days, 45 days, 56 days, 62 days respectively. On two occasions, 39 days and 53 days, first entry mechanisms mutants reaches noticable levels; however they become extinct due to small population size and stochastic fluctuation. At 65 days population the cargo is redesigned and overwhelming majority of the population returns to WT. The first cycle of redesign is shown in second and third panel. After the redesign, a similar sequence of events happens until the next redesign at 125 days. Qualitatively this redesign cycle is repeated for a total of 7 times. Subsequent to these events, the next mutant that arises is the mutant having two of its entry mechanism mutated (0,2). . This phenomenon of «skipping» an intermediate stage is observed in nature and is called stochastic tunneling. [2] Of course the intermediate population does exist, but only for a very short time and at a very low population. The tunneling is indicated by the short vertical bold red line at 434 day. While (0,2) takes higher portion of the population, at 438 day (0,1) and (1,0) mutants arise; however they are shortlived. The mutant (0,2) gives rise to (1,2) at 443 days, (1,2) gives rise to (2,2) at 448 days, (2,2) gives rise to the mutant having three mutated entry mechanisms (2,3) at 456 days and this mutant takes over the population at 460 days. (2,3) gives rise to fourth entry mechanism mutant (2,4). Eventually the fifth and final entry mechanism mutates at 463 days. Soon after final mutation there is a redesign at 463.46. This redesign drives back all strains back to either (0,4) or (0,5), this population cannot be rescued any further and the therapy fails at 463.67 days. B) Change of doubling time through time is plotted. The threshold of 56 minutes is reached at 65 days. Red lines for first two redesigns are plotted but the later ones are omitted for clear presentation. Redesign times are easily distinguishable by sudden jumps in doubling time. Cargo is redesigned at this time and doubling rate is recovered to per 420 minutes. This cycle repeats 6 more times. C) Relative populations of entry mechanism mutants are plotted with log scale. This representation enables us to spot the stochastic tunneling that happened in this trajectory. The first entry mechanism mutant stays low but fluctuates. Many times, the second entry mechanism mutates but eventually dies. At 410 days, the second entry mechanism mutant arises from the low single mutant and increases to take over the population. Soon after the second entry mechanism, the third mechanism fails at and dominates the population. After third entry mechanism, fourth and fifth entry mechanism fails very rapidly and therapy is halted at 460 days.

In Fig E, trajectory of evolution is simulated with parameters $\omega$=5 and 3 independent entry mechanisms. Fig E, panel A, shows the Muller plot. Therapy fails at 113.2 days with total of 10 redesigns. In Fig E, panel B, doubling time versus time is plotted. In Fig E panel C, the entry mechanism mutants are plotted with log scale. The first entry mechanism arises sporadically until 110 days, at this time it dominates the population. At 113.2 days the third entry mechanism mutant arises and the therapy fails.


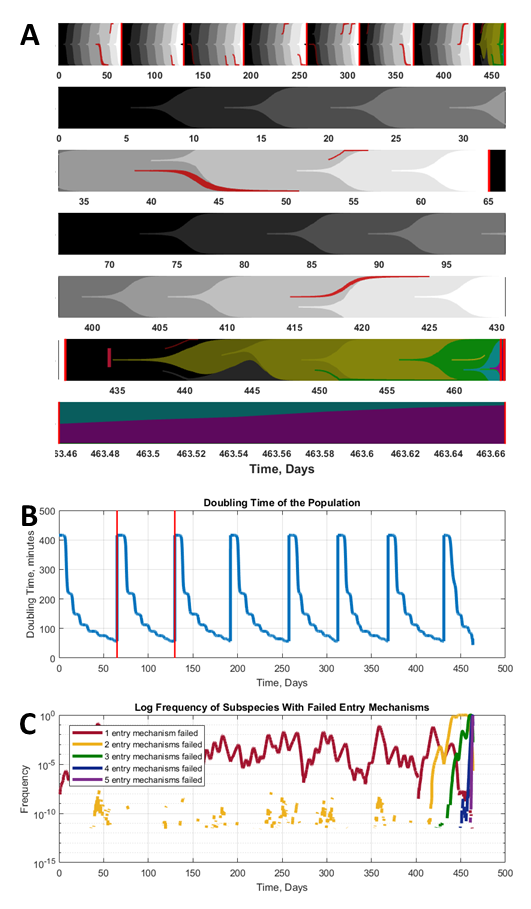


**Fig E:** **Sample Trajectory with Three Entry Mechanism and ω=5:**

A) The top panel shows the full trajectory, last panel is the last redesign cycle (111.4 days to 113.2 days), and the panels in between are shorter parts of the full trajectory for more detailed inspection. The vertical red lines mark the time of redesign. Therapy lasts for 113.2 days. In this trajectory cargo is redesigned 10 times. Until first redesign we see (0,1), (0,2) dominates the populations at 4 days, 7 days respectively. At 9 days (0,3) arises from (0,2) and increases until the redesign. First redesign is made at 11 days. The first cycle of redesign is also shown in first panel. After the redesign, the same sequence of events happens until the next redesign at 23 days. Similar redesigns cycles happen 6 times more. After the redesign at 91 days, the trajectory proceeds similarly to other cycles. However, there is a significant difference at the end of this cycle. At 103 days (2,1) mutant arises from (2,0) and does not die off until the redesign. After this redesign at 105 days, 10 percent of total population has a single-entry mechanism mutation. This population, (0,1), grows and gives rise to (1,1) at 107 days and it dominates the population at 107.5 days. (1,1) gives rise to two mutants (2,1) and (1,2) mutants. Both mutants survive until the redesign at 111.5 days. Double non-specific-mutant is present at the start of the cycle and quickly gives rise to (0,3) at 112.7 days which takes over the population beyond recoverability. B) Doubling time versus time is plotted. As usual doubling time starts at 420 minutes per generation. The doubling time decreases stepwise (growth rate increases) as mutants’ takeover. The threshold of 60 minutes is reached at 10 days. Cargo is redesigned at this time and doubling rate is recovered to per 420 minutes. Redesign is indicated with vertical red line. Red lines for first three redesigns are plotted but the later ones are omitted for clear presentation. Redesign times are easily distinguishable by sudden jumps in doubling time. This cycle repeats 10 more times, at last redesign cannot rescue the therapy. C) The entry mechanism mutants are plotted with log scale. The first entry mechanism arises sporadically until 110 days, after this time it dominates the population. At 113.2 days, the third entry mechanism mutant arises and the therapy fails.

In Fig F, trajectory of evolution is simulated with parameters $\omega$=5 and 5 independent entry mechanisms. Fig F panel A shows the Muller plot. The therapy lasts for 1179 days. In Fig F panel B, doubling time versus time into experiment is plotted. In Fig F figpanel C, the entry mechanism mutants are plotted with log scale. The first entry mechanism escape mutant arises sporadically until 1000 days, at which time it dominates the population. At 1100 days the second entry mechanism mutant arises and dominates the population. Soon after the fourth and yet faster the fifth entry mechanism fails.


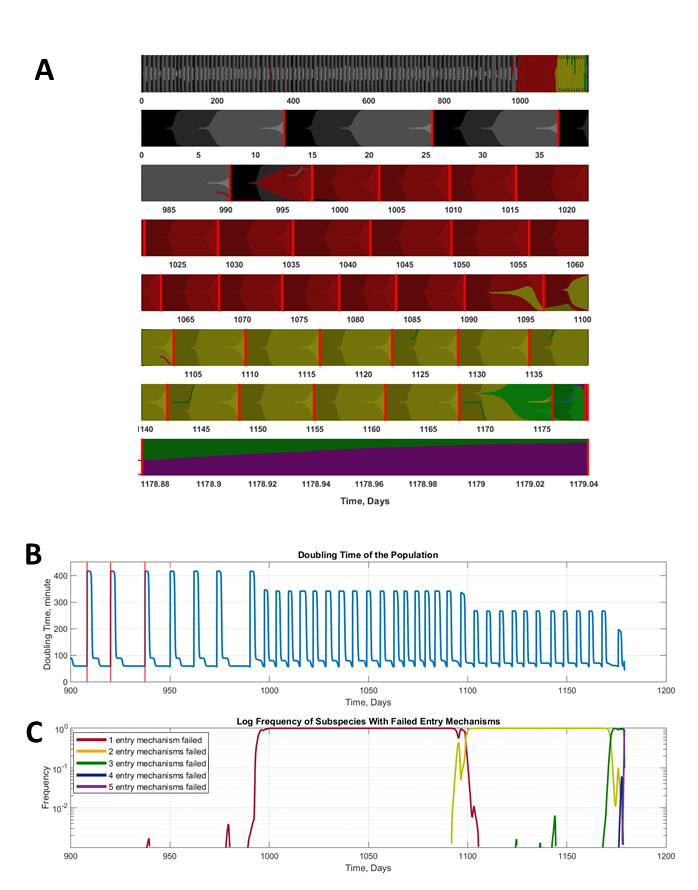


**Fig F:** **Sample Trajectory With Five Entry Mechanism and ω=5:**

A) The top panel shows the full trajectory, last trajectory is the last redesign cycle (1178.88 days to 1179.04 days), and the panels in between are shorter parts of the full trajectory for more detailed inspection. The vertical red lines mark the time of redesign. Therapy lasts for 1179.04 days. In this trajectory cargo is redesigned about 100 times. Until first redesign we see (0,1), (0,2) dominates the populations at 3.5 days, 7 day respectively. At 10.5 days (0,3) arises from (0,2) and increases until the redesign. First redesign is made at 12.5 days. After the redesign, same sequence of events happens, happens qualitatively similar until 991 days. After 991 days, WT, (0,0), gives rise to (0,1) at 992 days, (0,1) gives rise to (1,1) 92.5 days. (1,1) mutant increases rapidly and dominates the population at 996 days. At this point the overwhelming majority of the population has lost one of the entry mechanisms. At 997 days cargo is redesigned and the majority of the population is (0,1). The mutant, (1,1) arises from (0,1) at 999 days, and (2,1) arises from (1,1) at 102 days. The growth of (2,1) continues until the redesign at 1003 days. The same sequence of events that happened in the previous cycle occurs until the redesign at 1090 days. It is important to note the time interval between redesigns. When the system lost a single entry mechanism the interval was around 10 days, while the cycles where all entry mechanisms were intact had 13 days. After the redesign at 1090, (1,2) arises from (1,1) at 1098 days and takes over the population by 1100 days. Subsequently, (2,2) arises from (1,2) at 1102 days and the system passes the threshold for redesign at 1104 days. After the redesign at 1104 days, (1,3) arises from (0,3) at 1106 days and subsequently (2,3) arises, then another cycle of redesign is made. This cycle also continues until redesign at 1168 days. After 1168 fourth entry mechanism fails, and after the redesign at 1176 the fifth also is lost. After losing all entry mechanisms, the final redesign happens at 1178.88 days. At this point there are mutants who had neutralized all entry mechanisms and one who had neutralized 4. The mutant that neutralized all entry mechanisms quickly took over the population from 1178.88 to 1179.04, as also seen at last panel. The therapy ends at 1179.04. B) Doubling time versus time during experiment is plotted for times greater than 900 days. As usual doubling time starts at 420 minutes per generation. The doubling time decreases (growth rate increases) as mutants take over. The threshold of 60 minutes is reached at approximately 10 day intervals. Each time the threshold the cargo is redesigned doubling rate recovers.. At approximately 1000 days the population has neutralized one of the entry mechanisms, after which recovery of the therapeutic effectiveness after cargo redesign is less complete . With each subsequent neutralization of an entry mechanism the degree of recovery is less complete and the time until loss of an additional entry mechanism is accelerated, until the therapy finally fails. C) For the same computer experiment as B) the entry mechanism mutants are plotted with log scale, in order that small subpopulations can be seen.

**Mean hit-time calculations**

Calculating a mean hit-time for a continuous Markov chain is described in the textbook by Norris [4]. For every node, other than the target node, a linear equation is written. First term is the wait time at the current node, which is the reciprocal of the total rates going out from that node. The remaining terms consist of two parts, probability to jump to a node multiplied by mean hit-time to the target from that node. The equations related to calculations in the Fig E are given here:

$$k1 =\frac{1}{r1}+k2,$$

$$k2 =\frac{1}{r2+r4}+\frac{r2}{r2+r4}k3+\frac{r4}{r2+r4}k4,$$

$$k3 =\frac{1}{r3+r5}+\frac{r3}{r3+r5}k1,$$

$$k4 = 1/r6$$

General solution for k1 is,

$k1 =\frac{r1r2r6+r1r3r4+r1r3r6+r1r4r5+r1r5r6+r2r3r6+r2r5r6+r3r4r6+r4r5r6}{r1r6\left( r2r5+r3r4+r4r5 \right)}$ Equation (A)

**Limit of infinite redesign efficiency: the limit of r3 going to infinity**

$\lim_{r3\to\infty} k1=\frac{1}{r6}+\frac{1}{r4}+\frac{r2}{r1 r4}+\frac{1}{r1}$

Let’s assume r1 and r6 as 1

$\lim_{r3\to\infty} k1=2+\frac{1}{r4}+\frac{r2}{r4}$ Equation (B)

**Redesign efficiency (RE) versus Proposed Strategy (CS)**

We let,

$$r3=RE$$

$$r2=PS$$

$$r4=\frac{1}{PS}$$

$$r1,r5,r6=1$$

Then,

$k1=\frac{\left( RE+2 \right) PS^{2}+\left( RE+1 \right) PS+2 RE+2}{PS^{2}+RE+1}$ Equation (C)

**References**

[1] Barrick JE, Lenski RE. Genome dynamics during experimental evolution. Nature Reviews Genetics. 2013 Dec;14(12):827.

[2] Iwasa Y, Michor F, Nowak MA. Stochastic tunnels in evolutionary dynamics. Genetics. 2004 Mar 1;166(3):1571-9.

[3] Shaffer ML, Samson FB. Population size and extinction: a note on determining critical population sizes. The American Naturalist. 1985 Jan 1;125(1):144-52..

[4] Norris JR. Markov chains. Cambridge university press; 1998 Jul 28.
